# Immunoglobulin Y for Potential Diagnostic and Therapeutic Applications in Infectious Diseases

**DOI:** 10.3389/fimmu.2021.696003

**Published:** 2021-06-09

**Authors:** Lucia Lee, Kate Samardzic, Michael Wallach, Lyn R. Frumkin, Daria Mochly-Rosen

**Affiliations:** ^1^ Department of Chemical and Systems Biology, Stanford University School of Medicine, Stanford, CA, United States; ^2^ School of Life Sciences, University of Technology, Sydney, NSW, Australia; ^3^ SPARK at Stanford, Stanford, CA, United States

**Keywords:** immunoglobulin Y, IgY, chicken immunoglobulin, pathogen, viral (or virus)

## Abstract

Antiviral, antibacterial, and antiparasitic drugs and vaccines are essential to maintaining the health of humans and animals. Yet, their production can be slow and expensive, and efficacy lost once pathogens mount resistance. Chicken immunoglobulin Y (IgY) is a highly conserved homolog of human immunoglobulin G (IgG) that has shown benefits and a favorable safety profile, primarily in animal models of human infectious diseases. IgY is fast-acting, easy to produce, and low cost. IgY antibodies can readily be generated in large quantities with minimal environmental harm or infrastructure investment by using egg-laying hens. We summarize a variety of IgY uses, focusing on their potential for the detection, prevention, and treatment of human and animal infections.

## Introduction

**Figure 1:**
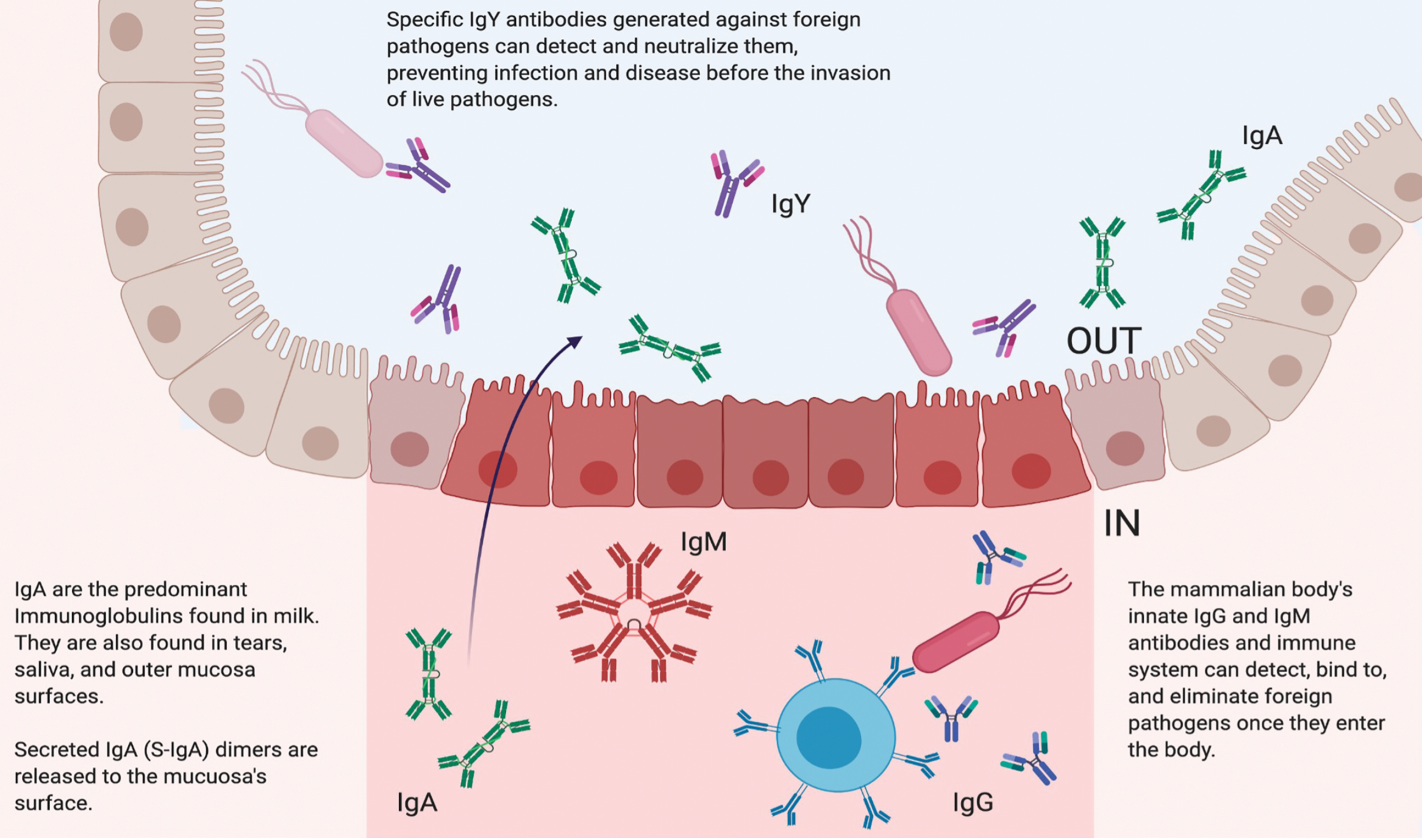
Biological Function of Different Immunoglobulins. In mammals, immunoglobulin G (IgG) is the main antibody in the circulating bloodstream. IgG antibodies are characterized by two antigen recognition sites that act as crucial components of the immune system. These sites are involved in the detection and subsequent elimination of foreign invaders that may also be circulating in the bloodstream. Mammalians also produce immunoglobulin M (IgM) antibodies, which are found in blood and lymph fluid and the first antibodies to appear in response to antigen exposure. Immunoglobulin A (IgA) antibodies are the main immunoglobulins found in milk, tears, saliva, and mucosa. IgA is characterized by a unique dimer structure and released to the outer mucosa surface. Immunoglobulin Y (IgY) is the main antibody found in birds, reptiles, and amphibians. Because of their unique structures, IgY antibodies are particularly resistant to low pH, high temperatures, and proteolysis. IgY can be effectively administered prophylactically and therapeutically to detect and neutralize pathogens without activating the host’s native immune system.

Immunoglobulin Y (IgY) is the main class of serum antibodies found in birds, amphibians, and reptiles (1). IgY, the avian homolog of immunoglobulin G (IgG) in humans (2), is the evolutionary ancestor of IgG (1) and shows significant sequence conservation (3). IgG and IgY are composed of two heavy and two light chains, each with constant and variable regions. The variable regions are critical for antigen recognition, whereas the constant regions provide the effector function. IgG and IgY are responsible for similar immune response functions (**Figure 1**) (1). Compared with IgG, IgY has a higher molecular weight (180 kDa vs. 150 kDa) and contains a larger heavy chain (65-68 kDa vs. 50 kDa) (1). In addition, IgY contains more carbohydrate sidechains (2 vs. 1), is more hydrophobic especially in the longer Fc portion of the molecule (3 constant domains in IgY vs. 2 in IgG), and is more resistant to proteolysis than its mammalian counterparts (4). IgY also retains 40% of its activity after incubation with trypsin or chymotrypsin for 8 hours (5).

In healthy humans, serum half-life is approximately 23 days for IgG, 5 days for IgM, and 6 days for IgA (6). Although the precise *in vivo* half-life of IgY is not well characterized in humans, active IgY antibodies were still present in the saliva of patients with cystic fibrosis in the morning after gargling with an anti-*P. aeruginosa* IgY solution the previous evening (7). In suckling pigs, IgY had a half-life of 1.85 days in the sera and 1.73 hours in the gastrointestinal tract (8). Similar to IgG, IgY is involved in opsonization, complement system activation, and most effector functions in the chicken (9). However, IgY is unable to bind to or activate the mammalian complement system, Fc receptors, and rheumatoid factors (10, 11).

IgY may provide a low cost (only a few cents to produce an oral or nasal dose), safe (due to its molecular structure, see below), and fast approach (produced in 5-8 weeks, including the time required for immunogen preparation) for rapid prophylaxis and therapeutic development against a variety of pathogens. In particular, passive immunoprophylaxis by surface treatment with IgY (e.g. *via* nasal spray, lozenges, etc.) may be an effective method of capturing pathogens before they enter the body. This approach may be especially attractive in limited-resource countries where

vaccination is unavailable, where new viral variants may evade immunity produced by vaccines or previous infection, or where immediate immunity is required (such as an outbreak in a nursing home).

Antiviral drug and vaccine development is lengthy and costly. For example, the estimated mean duration of clinical development was 6.4 years for 48 new antiviral drugs licensed for use in the United Kingdom between 1981 and 2014 (12). Because of the accelerated research, development, and Emergency Use Authorization during the coronavirus disease 2019 (COVID-19) pandemic, pharmaceutical companies have been able to obtain approval for therapeutics and vaccines within a dramatically shorter time and at a lower cost. Typically, scaling up the production of antiviral therapeutics is difficult, even for costs to manufacture new COVID-19 treatments (13). This is especially challenging in resource-limited-income countries due to the costly infrastructure required to develop, distribute, and store antiviral agents and vaccines. In contrast, IgY production may take as little as 2 months and occur on a community level through the inoculation of laying hens. These hens can produce eggs containing protective IgY antibodies that can be easily processed into a long-lasting and easy-to-store lyophilized product.

**Figure 2:**
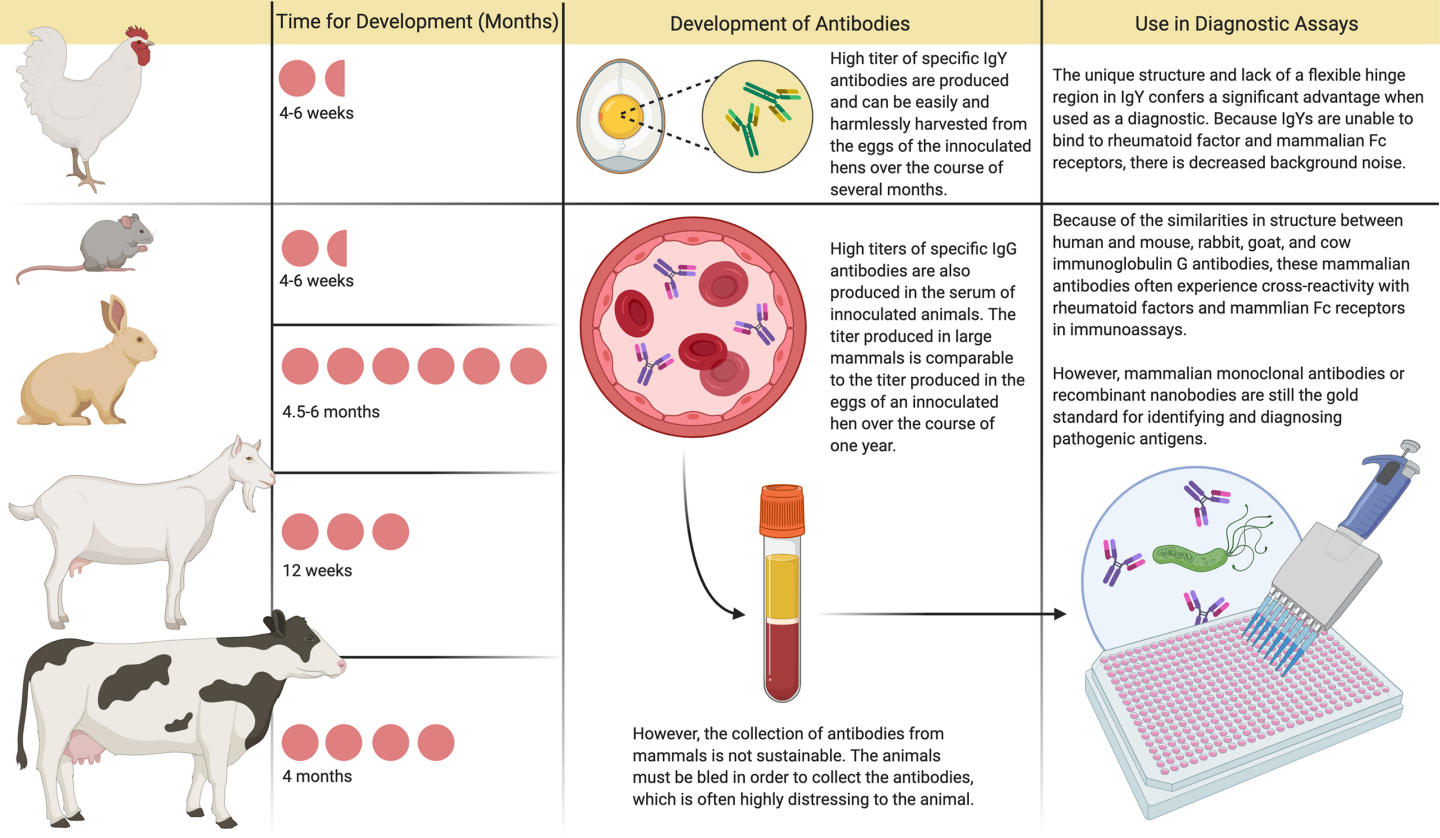
Immunoglobulin Y and Immunoglobulin G Antibody Development and Use in Diagnostic Assays. In animals raised for conventional antibody production, immunoglobulin G (IgG) is the main serum antibody collected. The amount of antibody that can be harvested from the blood in one setting is comparable to the amount of immunoglobulin Y (IgY) produced in the eggs of a commercial laying hen over a year. However, production of these conventional antibodies usually requires 2-6 months, and harvesting through bleeding is distressing to the animal. Currently, mammalian monoclonal antibodies are considered the gold standard for use in diagnostic assays, although they are prone to exhibit cross-reactivity and are costly to produce. In contrast, after inoculation with the desired antigen, hens produce specific antibodies in their eggs within 4-6 weeks. These IgY antibodies can be easily harvested and used in diagnostic assays, with lower background and cross-reactivity compared with current industry standards.

IgY used as prophylaxis can also be tailored to be highly specific and is not dependent on the host immune response (**Figure 1**). This latter feature may enable its clinical application for protecting a wide range of especially vulnerable patients, including the elderly, immunocompromised, and young children (14). In addition, in animals raised for conventional antibody production, IgG is the main serum antibody collected. In contrast, IgY antibodies can be easily harvested and used in diagnostic assays, with lower background and cross-reactivity compared with current industry standards using IgG in animals raised for conventional antibody production (**Figure 2**).

Here we provide a comprehensive overview of previous clinical applications of IgY in both humans and animals, including viral, bacterial, fungal, and parasitic infections.

## Diagnosis and Treatment of Viral Infections

### Detection and Neutralization of Coronaviruses: Severe Acute Respiratory Syndrome (SARS) Coronavirus and Middle East Respiratory Syndrome (MERS) Coronavirus

Severe Acute Respiratory Syndrome (SARS) is caused by SARS-associated coronavirus (SARS-CoV) and characterized by fever, chills, cough, pneumonia, and high infectivity. SARS was first identified in southern China in 2003 but quickly spread to over 30 countries, resulting in greater than 8000 cases and an estimated mortality rate of 15% (15). Middle East Respiratory Syndrome (MERS) is also caused by a coronavirus (MERS-CoV) and was first identified in humans in the Middle East in 2012; individuals infected with MERS showed similar clinical features to those infected with SARS (16). Because of the high infectivity and mortality caused by coronaviruses, prophylaxis to capture and neutralize the virus before it enters the nasopharynx using IgY against SARS-CoV or MERS-CoV could provide a means to curb the transmission and severity of disease. IgY against SARS-CoV or its spike protein neutralized the virus in infected Vero E6 cells (17, 18). Similarly, anti-MERS-CoV IgY neutralized MERS-CoV in infected Vero E6 cells, inhibited viral replication *in vitro*, and conferred protection when injected intraperitoneally to mice challenged with MERS-CoV (19). IgY against the nucleocapsid protein of SARS-CoV, which is shed in high amounts during the first week after infection, was found to be highly sensitive and capable of detecting the virus in serum and nasopharyngeal aspirate (20), indicating the potential for IgY-based diagnostic tools. When faced with highly contagious infectious diseases, such as those caused by coronaviruses, there may be several advantages to using IgY as a diagnostic tool (**Figure 2**) in addition to combating community transmission or providing passive immunization to exposed individuals.

### IgY Against SARS-CoV-2

Besides regulatory authorizations or approvals of COVID-19 vaccines, antivirals, and neutralizing antibodies in various countries, there remains a global need to develop additional safe, effective, easy-to-produce, and inexpensive treatments to prevent or reduce the risk of acquiring severe acute respiratory syndrome coronavirus 2 (SARS-CoV-2) infection (21, 22). This need is of heightened importance for health care and other at-risk service workers in regions where new variants of SARS-CoV-2 may increase contagiousness or evade immunity produced by vaccines or previous infection (23, 24) or where COVID-19 vaccination is unavailable. Despite the use of personal protective equipment and recommended practices, these groups are also vulnerable during the immediate period after active vaccination due to continued close contact with both asymptomatic and symptomatic individuals infected with SARS-CoV-2 (25).

**Figure 3:**
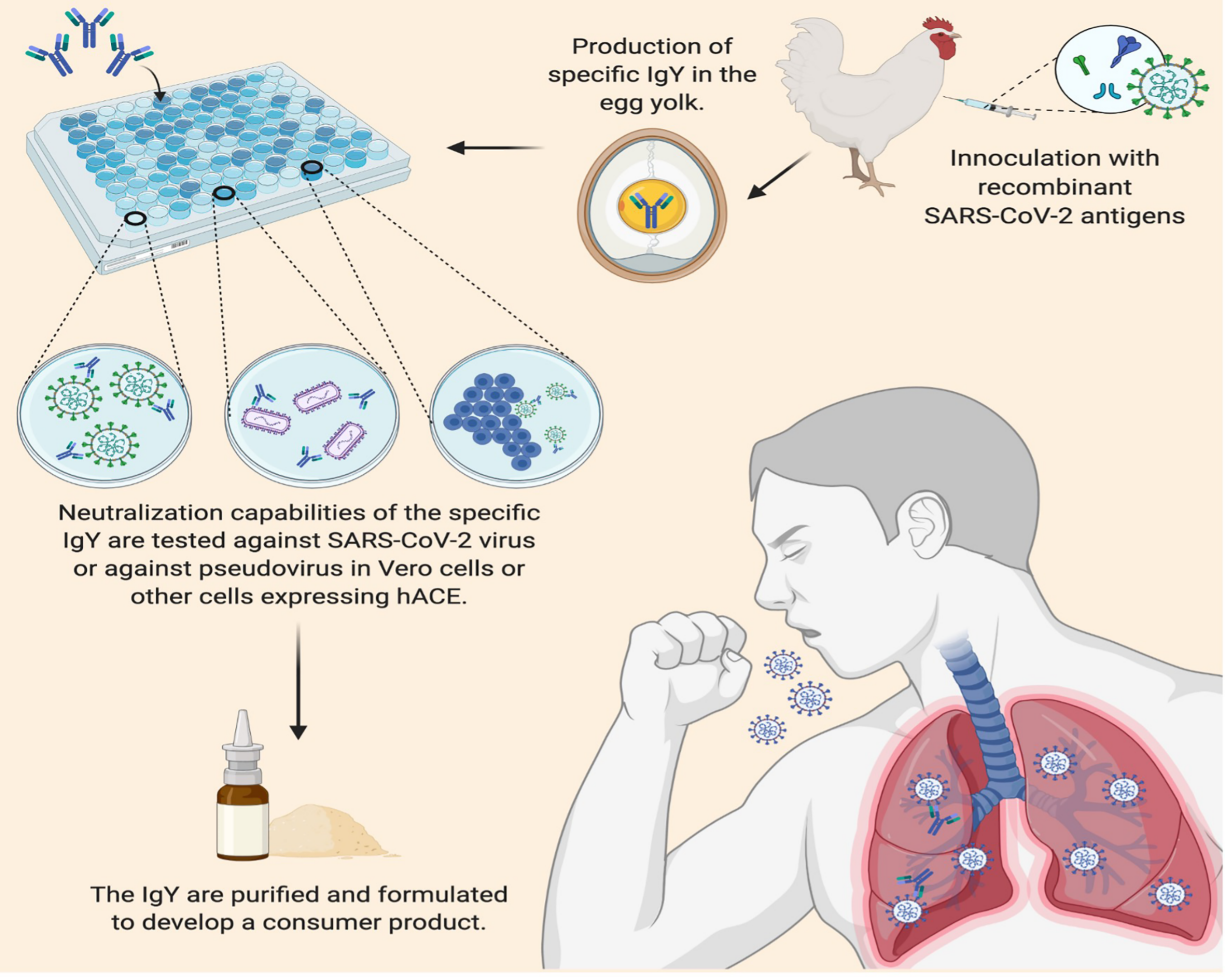
Applications of Immunoglobulin Y against SARS-CoV-2 to Inhibit COVID-19. IgY can be easily produced by inoculating egg-laying hens with recombinant SARS-CoV-2 antigens such as the spike protein on the viral envelope. Following a second inoculation, the yolk of the eggs laid by commercial hens contains 50-100 mg IgY per egg; up to 500 mg IgY per egg can be obtained from naïve, specific-pathogen-free hens. After harvesting the IgY from the egg yolks, the efficacy of IgY antibodies can be tested for neutralization activity before purification and processing into a lyophilized product to be evaluated in controlled trials as a potential (prophylactic) COVID-19 passive immunization.

Active vaccination requires the induction of an immune response that takes time to develop and varies depending on the vaccine and recipient. In contrast, passive antibody administration may provide immediate immunity to susceptible persons. However, passive immunization *via* parenteral administration with sera from recovered patients infected with SARS-CoV-2 requires the availability of a population of donors who have recovered from the disease and can donate convalescent serum, blood banking facilities to process the serum, assays to detect SARS-CoV-2 in serum, assays to measure viral neutralization, and virology laboratory support to perform these assays (26). Passive immunization with anti-SARS-CoV-2 IgY given topically may offer another passive immunization with several advantages: the known safety profile of IgY, applicability to a wide array of individuals, high yield per egg, and rapid mass production at a low cost given the large production of eggs for human consumption (27, 28) (**Figure 3**). In addition, anti-SARS-CoV-2 IgY applied superficially would not be expected to elicit antibody-dependent enhancement of infection due to its topical application.

Intranasal antibody prophylaxis is an especially effective means against multiple viral pathogens (29–32). Further, key characteristics of the mechanism of action of anti-SARS- CoV-2 IgY may be ideal for effective transient immunization while awaiting global COVID-19 vaccination: a) anti-spike-S1 IgY can neutralize or bind SARS-CoV-2 *in vitro* (33–35), b) superficially administered anti-SARS-CoV-2 IgY may bind to the spike protein on the surface of the virus, competing with the binding of the viral spike protein to the human angiotensin-converting enzyme 2 receptor to prevent cell entry and infection, c) anti-SARS-CoV-2 IgY may also agglutinate SARS-CoV-2 on the surface of the mucosa, thus preventing viral lateral motility and entry across the mucosa, and d) intranasal administration can deliver anti-SARS-CoV-2 IgY to the nasal passage and throat mucosa (through mucociliary clearance), the main routes of viral entry and replication. This approach may be especially valuable for immediate and short-lived protection in health care and other at-risk service workers in regions where new variants of SARS-CoV-2 may evade immunity produced by vaccines or where COVID-19 vaccination is unavailable.

### Detection and Treatment of Influenza Viruses

Influenza is a highly contagious infection of the respiratory tract that is caused by the influenza family of viruses. There are four types of influenza viruses, but types A and B most commonly cause seasonal influenza pandemics. Influenza is generally a self-limited illness but is a significant global concern due to complications such as pneumonia and organ failure that can lead to death, especially in vulnerable patients (36). Although novel seasonal influenza vaccines are developed annually, influenza viruses are extremely prone to antigenic drift and effective vaccines reduce the risk of flu illness by only about 50% (37, 38). Because of the limitations of vaccination and antiviral drugs, anti-influenza IgY may be an inexpensive and rapid prophylaxis that can be given *via* intranasal spray or drops to bind to numerous epitopes on the virus and thus prevent viral entry into the body (39). This approach may be practical for those where vaccination is unavailable or as a temporary measure (such as during travel) before or to supplement vaccination.

IgY developed against various influenza strains was effective in neutralizing the virus and protecting against infection. In vitro, IgY anti-H1N1 inhibited hemagglutination (39, 40) and infection of Madin-Darby canine kidney cells by binding specifically to virus neuraminidase and hemagglutinin (39, 41). In a mouse model of H1N1 infection, intranasally administered anti-H1N1 IgY reduced the viral titer and minimized damage to the lung tissue while conferring significant protection comparable to oseltamivir (39), a neuraminidase inhibitor approved to treat acute uncomplicated influenza and for prophylaxis of influenza. Similar results were also observed using IgY developed against inactivated H3N2, H5N1, and influenza B viruses; these neutralized the virus *in vitro* and protected challenged mice when administered intranasally at (42–44). In addition, anti-H1N1 and anti-H5N1 IgY exhibited cross-reactivity to other influenza A strains (40, 41, 43).

IgY has also been developed against specific influenza proteins. For example, IgY has been designed to target the influenza matrix 2 protein, which is secreted in high concentrations during infection and can be used in the diagnosis and treatment of influenza. In immunoassays, IgY anti- matrix 2 protein were specifically bound to the influenza virus and caused aggregation when coupled to a latex nanoparticle (45). IgY generated against the highly conserved nucleoprotein of influenza A strains bound specifically to the viral protein and virus in infected Madin-Darby canine kidney cells (46). Therefore, IgY may prove useful for diagnosis and prophylaxis against various circulating or novel strains of human, avian, or swine influenza viruses.

### Treatment of Hepatitis B

Hepatitis B viruses have a narrow range of host specificity (47). Similar to the human hepatitis B virus (HBV), duck HBV infection can lead to chronic hepatitis, fibrosis, cirrhosis, and hepatocellular carcinoma (48). In eggs laid by ducks that were immunized against duck HBV, significant titers of specific IgY were found in the yolk (49, 50). Also, uninfected treated ducks were protected against the virus, and treated carriers had decreased serum and liver levels of duck HBV (51, 52). Despite the availability of an effective vaccine, rates of vaccination in humans are poor and immunotherapies may be unavailable in resource-limited settings, or not tolerated. IgY may also warrant evaluation as a therapeutic for the treatment of postexposure HBV infection in humans where standard treatments are not readily available.

### Treatment of Rotavirus Group A

Rotaviruses cause gastrointestinal and diarrheal diseases, resulting in high rates of mortality in various animal species and severe watery diarrhea and vomiting in infants and young children (53). Although vaccines and symptomatic treatments are available, there is a special need for effective control strategies to eliminate rotavirus infections in animal settings to protect herds (54); IgY against the bovine Group A Rotavirus VP8 capsid protein may be one such avenue (55, 56). In challenged calves, specific IgY against bovine rotavirus, combined with milk formula or distilled water, increased the response of the mucosal antibody-secreting cells, reduced viral shedding, decreased the severity and duration of diarrhea, and minimized body weight loss (44, 57–60). Furthermore, an orally administered solution of specific IgY against the MO strain conferred complete or significant protection against strains of rotavirus in later infected BALB/c mice (61, 62). With combination IgY treatment against the G1, G4, and G9 serotypes, protection was also observed in infected mice administered feed containing specific IgY, as well as a marked reduction in viral load, histopathological damage, and diarrhea (63). Decreases in duration and extent of diarrhea were observed in infected mice treated with an orally administered solution of specific IgY (4), a Rotamix IgY treatment (64), or feed containing IgY in phosphate-buffered saline (65). In a neonatal gnotobiotic piglet disease model, a 4.8% milk solution supplemented with anti-human rotavirus IgY increased IgG and IgM antibody response against the virus, decreased viral shedding, and protected against diarrhea (66).

Rotavirus is the most common cause of severe diarrhea in children and outbreaks can occur in both vaccinated and unvaccinated children (67). When administered to pediatric patients who tested positive for rotavirus infection, orally administered treatments of 20% IgY sachets (64) or 4 doses of 10 g of IgY powder (68) significantly reduced the time for viral clearance in the feces, volume and duration of oral rehydration, intravenous fluid administration, duration of diarrhea, and recovery time. Wang et al. (69) conducted a meta-analysis involving 2626 infants with rotavirus diarrhea from 17 randomized clinical trials. Among these infants, 1347 received anti-rotavirus IgY taken orally and 1279 received conventional treatment. Anti-rotavirus IgY treatment was significantly more effective than conventional treatment. These preliminary data demonstrate the utility of IgY as an antiviral therapy for infantile rotavirus enteritis.

### Treatment of Zika Virus

In 2016, a global health emergency was declared by the World Health Organization after the observation of hundreds of thousands of infections by the Zika virus. Zika virus is transmitted by Aedes mosquitoes (70). The illness is characterized by fever, rash, arthritis (71), and less commonly, Guillain-Barré syndrome (72). In pregnant women, Zika virus infection may cause severe birth defects including microcephaly (73, 74). There is no effective drug or vaccine for Zika virus infection in part because of antibody-dependent enhancement, a phenomenon in which prior infection results in virus-specific antibodies enhancing replication of virus into monocytes/macrophages and granulocytic cells through interaction with Fc or complement receptors (75). IgY against the Zika virus was able to neutralize the virus *in vitro* at a concentration of 25 µg/mL (76). Furthermore, intraperitoneal injection with 1 mg of specific IgY protected 3-week-old IFNAR−/− mice that received a lethal challenge of Zika virus without inducing antibody-dependent enhancement (76). Zika virus-specific IgY may warrant further evaluation as passive immunotherapy, with caution for the potential to elicit an allergic response.

### Detection and Treatment of Dengue Fever

Dengue fever, dengue hemorrhagic fever, and dengue shock syndrome are tropical, mosquito-borne diseases that are caused by infection with one of four dengue virus serotypes (77). In the last 50 years, dengue virus-associated diseases have re-emerged, causing millions of infected cases and tens of thousands of deaths annually (78). Although there is a commercially available vaccine to treat dengue fever, the World Health Organization recommends only vaccinating seropositive individuals who have a history of dengue virus infection, or by age 9 years in areas where the infection is prevalent (79). Currently, IgG-coupled enzyme-linked immunosorbent assays (ELISAs) are used for serological testing for dengue virus infection (79), suggesting that IgY can also be used in a diagnostic test for this disease.

Specific IgY has been developed against nonstructural protein 1, which is secreted by the dengue virus during infection and is detectable for up to 9 days after infection (80, 81). IgY anti-nonstructural protein 1 that is highly specific can neutralize the virus in immunoassays (82–84). Specific IgY generated against dengue virus serotype 2 also demonstrated a similar ability to neutralize the virus (85). In vivo, IgY antibodies given at a dose of 150 μg (82) or 1 mg through intraperitoneal injection (85) protected mice lethally challenged with dengue virus, suggesting that specific IgY may be used as a treatment of dengue virus-associated diseases in humans provided that a severe immune response (e.g., anaphylactic shock) to the IgY will not be generated (86). IgY antibodies are not likely to lead to immune amplification of dengue virus infection, unlike IgG where enhanced viral uptake *via* IgG-bound dengue virus enters into monocytes/macrophages *via* Fc receptors (87).

### Treatment of Hantavirus Pulmonary Syndrome

Hantavirus Pulmonary Syndrome (HPS) is a rare, severe, and potentially fatal respiratory disease caused by infection with hantaviruses (most commonly, Andes virus) (88, 89). Infection is believed to occur primarily through inhalation or ingestion of rodent feces, urine, and saliva, or by rodent bites. Person-to-person transmission is also recognized. The mortality rate is estimated at 38% (90). There are no current immunotherapies, antiviral treatments, or vaccines to treat HPS (91). Specific IgY developed after immunization of ducks with Andes virus neutralized the virus *in vitro* (92, 93) and protected Syrian hamsters administered a dose of 12,000 neutralizing antibody units/kg through intranasal delivery (92) or 20,000 neutralizing antibody units/kg through subcutaneous injection (93) after receiving intramuscular and intranasal challenge. This suggests that IgY may warrant evaluation as a treatment following infection with the Andes virus to prevent the onset of HPS, especially in settings of clustering of cases.

### Treatment of Ebola Virus Infection

Ebola virus infection is rare but results in high mortality rates in humans and nonhuman primates (94). Ebola virus also can persist in survivors and relapse has been documented. In addition to supported care, two monoclonal antibody treatments and a vaccine have been approved (94).

Anti-Ebola virus IgY was harvested from hens immunized with a recombinant vesicular stomatitis virus vector encoding Ebola virus glycoproteins. Anti-Ebola virus IgY was then evaluated in newborn Balb/c mice challenged with a lethal dose of Ebola pseudovirus 2 or 24 hours after infection (95). Animals receiving a high dose of anti-Ebola virus IgY showed complete protection, while the low dose group showed partial protection. All mice receiving naïve IgY (i.e., from hens not immunized with Ebola glycoproteins) died. Zhang et al. note that because Ebola epidemics typically occur in impoverished hot African areas where electricity and cold-chain storage may be limited, advantages of low-cost mass production and avoidance of antibody-dependent enhancement may make anti-Ebola virus IgY an especially novel approach that warrants further investigation (95).

## Viruses of Specific Interest in Diseases of Livestock

### Treatment of Infectious Bursal Disease

Infectious bursal disease is a highly contagious avian immunosuppressive illness that is caused by the infectious bursal disease virus (IBDV). IBDV infection in bursal B cells of lymphoid organs can lead to cell destruction, suppression of the immune system, secondary infection, and death (96). Despite the development of various diagnostic methods as well as a recombinant DNA-IBDV vaccine to treat this disease (97), IBD remains a significant problem in the poultry industry due to the emergence of new variants (98). Anti-IBDV IgY can protect against infection, with decreased mortality, postmortem lesions, bursal tissue, and body weight loss in young broiler chicks lethally challenged with IBDV (99–101). This protective effect was most significant when specific IgY was combined with the live intermediate IBDV vaccine (D78) (99).

### Detection and Treatment of Reovirus

In poultry, the most frequent reovirus-associated disease is arthritis with malabsorption syndrome, immunosuppression, pericarditis, myocarditis, and osteoporosis as other common features (102, 103). Although several commercial vaccines have been developed against avian reovirus, it is difficult to detect and treat infected flocks with multiple or novel strains of circulating reovirus (104). Specific IgY against avian reovirus in infected birds displayed a high sensitivity to the virus, detected the presence of the virus in contaminated tissue, and neutralized the virus in BHK-21 cells without binding to heterologous viral strains (105). Reovirus is also a growing problem in aquaculture, causing high mortality rates of swimming crabs. Use of anti-swimming crab reovirus IgY effectively detected the virus in contaminated samples with high consistency, suggesting potential benefit in reovirus-associated disease outbreaks in aquaculture (106).

### Treatment of Bovine Respiratory Syncytial Virus

Bovine respiratory syncytial virus is a pneumovirus in the Paramyxovirus family that afflicts young calves and is difficult to diagnose and treat due to its lability and poor growth in cell culture (107). However, IgY against bovine respiratory syncytial virus specifically recognized and neutralized the virus *in vitro* when analyzed in dot blot and virus neutralization assays (108). Because of the success of IgY production and activity *in vitro*, IgY may be a novel prophylactic treatment to combat bovine respiratory disease in infected calves.

### Treatment of Bovine Leukemia Virus

Bovine leukemia virus (BLV) is a retrovirus that causes enzootic bovine leukosis, a chronic and slow-developing disease in cattle (109). While most infected animals remain asymptomatic, others can develop lymphosarcoma or persistent lymphocytosis.

BLV can easily be transmitted through birth and contaminated colostrum, milk, blood, exudates, and tissue (110). Currently, methods to minimize BLV transmission include careful herd management (110) given the unavailability of effective antiviral drugs and vaccines (111). IgY antibodies generated against the whole virus or the p24 core protein specifically bound to BLV particles (in an infected cell line), purified p24, and supernatants from *ex vivo* cultures of peripheral blood mononuclear cells from naturally infected animals (111). IgY against BLV may warrant evaluation as a passive immunization against this virus for enzootic bovine leukosis.

## Treatment of Bacterial Infections

Besides use as therapeutics, IgY used prophylactically may provide passive immunization to the treated host through neutralization of bacterial toxins, prevention of adhesion to host cells, and inhibition or destruction of bacterial enzymes (112). Here we review the use of IgY as treatments focused on bacteria mainly of the pulmonary, gastrointestinal, and skin systems.

### Lung Infections

#### Treatment of *Pseudomonas aeruginosa* in Cystic Fibrosis

Cystic fibrosis is a common, hereditary, and life-threatening disease associated with damage to the lungs, pancreas, and male sex organs (113, 114). Patients with cystic fibrosis are especially prone to debilitating chronic lung infections caused by bacteria such as *Pseudomonas aeruginosa* (115). Due to a fear of developing antibiotic-resistant strains, alternative treatments to chronic antibiotic therapy have been studied, including the use of IgY against *P. aeruginosa* as a method of passive immunization. Anti-*P. aeruginosa* IgY significantly increased the neutrophil-mediated respiratory burst and subsequent bacterial killing of *P. aeruginosa in vitro* (116, 117). Anti-*P. aeruginosa* IgY also inhibited murine pneumonia when administered intranasally as evidenced by reduced bacterial burden, inflammatory cytokines, inflammation of the lung tissue, and clinical symptoms, an effect enhanced by pretreatment with azithromycin (118). The benefit of specific IgY anti-*P. aeruginosa* is believed to be against the flagellin protein implicated in the motility, adhesion, and inflammation of *P. aeruginosa* (119).

In 17 patients with cystic fibrosis, prophylactic continuous oral treatment (solution gargled for 2 minutes and swallowed in the evening) with specific IgY against *P. aeruginosa* to prevent pulmonary infections for up to 12 years (114 patient-years) showed significant reduction in *P. aeruginosa* infections compared with 23 cystic fibrosis control patients, with no adverse events (120).

A randomized, double-blind, placebo-controlled Phase 3 trial of 164 patients age 5 and older with cystic fibrosis was conducted at 47 European sites from 2011 to 2015 to evaluate treatment with oral anti-*P. aeruginosa IgY* gargling solution (n=83) vs. placebo (n=81). Study drug was to be gargled and then swallowed once daily in the evening. Study duration (and primary outcome measure) was until the next *P. aeruginosa* infection was diagnosed or 2 years, whichever came first. There was no significant difference between treatment groups in time to first recurrence of *P. aeruginosa* infection (median, 26.3 months for IgY-treated group) or secondary endpoints of number of exacerbations, number of days of illness, and use of antibiotics (121). Despite findings of no efficacy, this study provided an excellent safety database. A total of 1972 adverse events (AEs), mostly mild in severity, were reported, 989 of which were in the placebo group and 980 in the IgY group. The incidence of AEs was also similar between the two groups. The most commonly reported AEs were abdominal pain, vomiting, pyrexia, nasopharyngitis, and upper respiratory tract infection. No deaths occurred. Only 5 AEs in the IgY-treated group and 20 AEs in the placebo group (none serious) were judged to be related to study drug (121).

#### Treatment of Mycobacterium Tuberculosis

*Mycobacterium tuberculosis* (MBTC) is responsible for the development of tuberculosis, a potentially fatal respiratory disease that also can cause extrapulmonary disease (e.g., of the urinary system). MBTC is increasingly becoming more difficult to treat due to antibiotic resistance (122). In a rat peripheral blood mononuclear cell model, administration of high concentrations of IgY anti-MBTC increased interleukin-2 and interferon expression (123). IgY against MBTC may warrant evaluation for use in combination with other immunotherapeutic treatments of tuberculosis (124). In addition, IgY may also be of interest as a novel treatment of pulmonary nontuberculous mycobacteria, regarded as more challenging to treat because of frequent antimicrobial intolerance, toxicities, resistance, and drug-drug interactions (125).

#### Treatment of Acinetobacter baumannii

*Acinetobacter baumannii*, a gram-negative bacterium, is the cause of nosocomial infections and outbreaks in hospitals worldwide, such as sepsis, urinary tract infections, pneumonia, or surgical wound infections. Due to its resistance to desiccation and antimicrobial agents, *A. baumannii* is associated with significant mortality, costs, and hospital stays, particularly in vulnerable patients (126). In a mouse model of *A. baumannii*-associated pneumonia, intraperitoneal anti-*A. baumannii* IgY specific to pan-drug-resistant strains reduced levels of inflammatory cytokines, lung inflammation, and mortality (127). Similar results were also seen with intraperitoneal injections of 40 μg of IgY developed against the inactivated whole-cell or outer membrane proteins of *A. baumannii*, which protected against nasally challenged mice, possibly by inhibiting bacterial adhesion (128).

### Oral and Gastrointestinal Infections

#### Treatment of *Vibrio*

The genus *Vibrio* is responsible for seaborne diseases that can cause illness in aquatic animals raised for consumption, as well as foodborne infections and gastroenteritis in humans who consume raw or undercooked seafood (129). IgY may offer promise for diagnosing and treating virulent *Vibrio*-associated diseases in aquaculture. In the past, *Vibrio* treatment relied on vaccination and antibiotics, which may cause unintentional side effects, antibiotic resistance, and the buildup of toxins (130, 131).

IgY has been tested against *Vibrio alginolyticus*, which causes infections and high rates of mortality in abalone (132–134). When incorporated into the feed, IgY anti-*V. alginolyticus* significantly increased the survival of small abalones challenged by *V. alginolyticus* (135). Similar results were obtained using IgY against *Vibrio anguillarum* in rainbow trout (136), ayu fish (137, 138), half-smooth tongue sole (139), shrimp (140), and carp (141).

*Vibrio parahaemolyticus*, which causes acute hepatopancreatic necrosis and death in white shrimp in aquacultures (142), can cause serious foodborne illness in humans. Anti-*V. parahaemolyticus* IgY inhibited bacterial growth in liquid media (143, 144), decreased bacterial load, and increased survival rates in *V. parahaemolyticus*-challenged white pacific shrimp (144), suggesting its potential use to prevent foodborne illness in humans. Another strain, *V. harveyi*, which decreases health and survival in white shrimp (145), is inhibited through agglutinating IgY antibodies (146). Anti-*V. harveyi* IgY, incorporated into feed before being challenged with virulent *V. harveyi*, also increased levels of jejunioxyhemocyanin, prophenoloxidase, lysozyme, phagocytosis, and bacterial agglutinin, as well as augmented coagulase activity and intracellular superoxide anion production in treated Indian white shrimp (147). The potential value of anti-*V. paraphaemolyticus* IgY to prevent foodborne illness was tested in mice; 300 µg/ml IgY administered by oral gavage decreased the levels of bacteria in the feces and increased survival compared to vehicle-treated infected mice or mice treated with non-specific IgY (148).

Much research has been done to address *Vibrio cholerae*, which causes cholera in humans. Cholera is a global and life-threatening gastrointestinal disease characterized by severe diarrhea (149). Through the consumption of *V. cholerae*-contaminated food and water, hundreds of thousands of infections occur every year, especially in developing countries (150). Current treatments of *V. cholerae* include rehydration and vaccines, although their efficacy is limited (151). The finding that orally administered anti-*V. cholerae* IgY can prevent and treat cholera in suckling mice (151) and that only 2.5 micrograms protected against cholera in these animals (152) is encouraging.

#### Treatment of Helicobacter pylori

*Helicobacter pylori* (*H. pylori*) is a bacterial cause of gastritis, gastric ulcers, and gastric cancer (153). Because of the development of resistance to antibiotics, treatment of *H. pylori* requires the incorporation of multiple antibiotics. *H. pylori* exhibits resistance to metronidazole (78%), levofloxacin (56%), multidrug treatments (53%), and clarithromycin (31%) (154). Thus, orally administered immunoglobulins, and particularly IgY, have been suggested as an alternative approach to treat *H. pylori*-related infections because they limit the development of antibiotic resistance. IgY has been developed against *H. pylori* urease (HPU), a protein likely required for bacterial adhesion to mucin or the surface of epithelial cells in the gastric mucosa (155).

In animal models, a chow diet containing 25 mg anti-HPU IgY/g and 0.16 mg famotidine/g reduced *H. pylori* activity in infected Mongolian gerbils and prevented colonization of *H. pylori* in the gastrointestinal tracts of uninfected controls (156). In the Mongolian gerbil model, anti- *H. pylori* IgY reduced inflammation, neutrophil and leukocyte infiltration, and gastric mucosal injury by interfering with the adhesion of *H. pylori via* its urease (157). A similar anti-inflammatory effect and reduction of *H. pylori* in the gastric mucosa were also observed in C57BL6/j mice treated with 60 mg of anti-*H. pylori* urease C IgY in either powder form or dissolved in phosphate-buffered saline (158). Importantly, orally administered IgY (100- 500 mg) in male C57BL/6 mice were more efficacious in eliminating *H. pylori* compared to treatment with the commonly used proton pump inhibitor pantoprazole (159). IgY against other *H. Pylori* antigens with good efficacy includes IgY against the outer inflammatory protein (160, 161), the neutrophil-activating protein (162), and the native or recombinant VacA protein l (163, 164).

An egg yolk powder dietary supplement containing IgY anti-HPU administered to a cohort of asymptomatic *H. pylori*-positive patients reduced the levels of *H. pylori* and aided the treatment of *H. pylori*-associated gastritis, with no side effects reported (165). In a study of 42

*H. pylori*-positive subjects, drinkable yogurt fortified with anti-HPU IgY significantly suppressed *H. pylori* infection and was well tolerated with no adverse effects (166).

#### Treatment of Porphyromonas gingivalis

*Porphyromonas gingivalis* (*P. gingivalis*), which causes biofilm on teeth (167), is quite resistant to the host immune response and leads to inflammation and bone loss associated with periodontitis (168). Anti-*P. gingivalis* IgY against gingipains, a protein family released by *P. gingivalis*, inhibited attachment of the bacteria in cultured human epithelial cells (169). In addition, IgY against a 40 kDa outer membrane protein prevented the aggregation of *P.gingivalis* with *Streptococcus gordonii*, another bacterial strain implicated in periodontitis (170). Furthermore, IgY-anti-*P. gingivalis* in dry feed or administered as an ointment in a dog model reduced biofilm formation and inflammation (171). Sublingual application of IgY anti-*P. gingivalis* in five periodontitis patients reduced the levels of the bacteria and gum bleeding (172).

#### Treatment of Prevotella intermedia

Like *Porphyromonas gingivalis*, *Prevotella intermedia* also causes gingivitis and other periodontal diseases (173) and can be associated in humans with systemic diseases such as diabetes mellitus (174), respiratory illnesses (175), cardiovascular disease (176), ischemic stroke (177), osteoporosis (178), and risk of low birthweight preterm pregnancies (179). In rat models challenged with *P. intermedia* on gingivae, IgY anti-*P. intermedia* treatment protected against gingivitis by decreasing gingival index, plaque index, bleeding on probing, white blood cell counts, and local inflammation typically associated with periodontal disease (180). Because of the success of anti-*P. intermedia* IgY in rat models, as well as the general challenge due to increased resistance to antibiotics, IgY treatment may provide an alternative in humans.

#### Treatment of Solobacterium moorei

*Solobacterium moorei* causes oral halitosis, periodontitis, and gingivitis (181, 182). *S. moorei* is susceptible to common antibiotics such as penicillin, vancomycin, and moxifloxacin (183). Specific IgY inhibited bacterial growth in liquid media and biofilm formation *in vitro* (184). In a mouse model challenged with *S. moorei*, 20 to 40 mg/ml of specific IgY decreased bacterial counts in the oral cavities of treated animals (184). Benefit in humans has not been determined.

#### Treatment of Fusobacterium nucleatum

*Fusobacterium nucleatum* is one of many pathogenic bacterial strains that contributes to halitosis and periodontitis (185). Although available treatments include chemical antiseptics, antimicrobials, and mechanical therapy (186), efficacy is limited by poor compliance and the development of antibiotic resistance. IgY anti-*F. nucleatum* has been suggested as an immunotherapeutic alternative to mediating the development of *F. nucleatum* and other bacteria in the oral cavity. In a periodontitis rat model, IgY anti-*F. nucleatum* inhibited the development of volatile sulfurous and odorous compounds and decreased the malodor index, levels of anti-inflammatory cytokines, and alveolar bone loss, while aiding periodontal restoration (186).

#### Treatment of Streptococcus mutans

*Streptococcus mutans (S. mutans)* is the main odontopathogen implicated in the development of dental carries in humans (187). Although most antibiotic treatments of *S. mutans* are effective, resistance to penicillin, erythromycin, amoxicillin, clindamycin, and lincomycin is common (188). IgY developed against *S. mutans* is highly stable and cross-reacts with other serotypes, including *S. salivarum* (189–191). IgY against *S. mutans* inhibited *in vitro* bacterial growth, biofilm development, and binding to bacterial adhesion proteins, and inducing agglutination of *S. mutans* (189, 191, 192). Rats exposed to *S. mutans* and fed a caries-inducing diet had reduced dental caries when lyophilized anti-*S. mutans* IgY was incorporated into their feed (193, 194), topical gel (195), or chitosan-enriched soy milk (192).

The efficacy of anti-*S. mutans* IgY in humans has also been evaluated. IgY anti-*S. mutans* incorporated into a mouth rinse significantly reduced levels of *S. mutans* in saliva within 4 hours, and in plaque within 7 days (189). In comparison to commercial toothpaste, anti-S. *mutans* IgY toothpaste reduced levels of S. *mutans* more quickly and colonization was suppressed as long as 2 weeks after discontinuation (196). Similar results were also observed with the administration of lozenges containing IgY against S. *mutans* glucosyltransferase, which significantly decreased salivary levels of *S. mutans* after 5 days (197).

#### Treatment of *Salmonella*

The *Salmonella* species, particularly *S. typhimurium* and *S. enteritidis*, are human and chicken pathogens (198, 199). *Salmonella*-specific IgY inhibited bacterial cell growth (200) by binding to and structurally altering antigens on the surface of the bacterium (200) or by causing bacterial agglutination (201). In a human epithelial Caco2 cell model, IgY anti-*Salmonella* antibodies also prevented the adhesion of the bacterium to cells (202).

IgY anti-*S. typhimurium* and IgY anti-*S. enteritidis* exhibited significant cross-reactivity (200) and agglutination (201, 203), which indicates that IgY against a specific *Salmonella* serovar may be useful in treating a broad range of different *Salmonella* strains. IgY anti-*S. typhimurium* reduced immune cell recruitment and cytokine release in a mouse model infected with these bacteria (204). A combination treatment of a probiotic with IgY anti-*S. enteritidis* also decreased colonization and fecal shedding in young, market-aged broiler chicks challenged with *S. enteritidis* (205), indicating the additional potential benefit of using IgY anti-Salmonella antibodies in animals consumed as food.

#### Treatment of Clostridium difficile

*Clostridium difficile* (CD*)* is a cause of morbidity due to diarrhea and mortality due to inflammation of the colon, especially in the elderly, immunosuppressed, and after chronic antibiotic use. This serious condition has been increasing in incidence. IgY anti-CD has shown promise for patients based on animal studies (206). For example, 0.5 mg of IgY against the CD’s FliD colonization-associated factor administered by gavage prevented the adhesion of CD and significantly enhanced survival rates in CD-challenged Syrian hamsters (207). Oral gavage treatments of 0.6 mg of anti-CD spore IgY delayed diarrhea onset and reduced spore adhesion to intestinal cells in mouse models, especially when coupled with an existing antibiotics treatment such as vancomycin (208). IgY anti-CD toxin A and B neutralized toxins and prevented recurrent infections in a hamster model (209). Delivery of IgY anti-CD toxins to the colon instead of the upper gastrointestinal tract was enhanced when IgY was coated on microbeads (210) or encapsulated in chitosan-Ca pectinate microbeads (211) in a rat model.

#### Detection and Treatment of *Campylobacter jejuni*

The *Campylobacter* species, in particular *Campylobacter jejuni*, is the most common cause of gastroenteritis in humans worldwide (212). Most human *C. jejuni* infections are caused by the consumption of contaminated poultry. However, *C. jejuni* seems to have a commensal relationship with chickens while acting as a pathogen in humans (213). Because of the use of growth-promoting antibiotics in the meat-producing industry, there is a rise in antibiotic-resistant *Campylobacter* strains (214). IgY against *C. jejuni* may provide an alternative to antibiotic use (215, 216). Because of its high specificity and limited cross-reactivity, IgY anti-*C. jejuni* also can provide a highly accurate method to detect food contamination with *C. jejuni* (217, 218).

To reduce the spread of *C. jejuni* from poultry to humans, IgY has been administered as a passive vaccine to chickens. IgY against *C. jejuni* has prophylactic and therapeutic effects through its ability to decrease overall and fecal bacterial levels in *C. jejuni*-challenged chickens (219–221). Similar results were also observed using IgY against *C. jejuni* adhesins and flagellins, which significantly reduced caecal colonization by *C. jejuni* and initiated the production of *C. jejuni* specific antibodies, although these may not play a role in protection (222, 223). Administration of anti-*C. jejuni* IgY also resulted in a significant reduction in transmission of *C. jejuni* to non-inoculated birds (220), without altering the microflora of the intestinal tract (221).

### Sepsis

#### Detection and Treatment of *Escherichia coli*

*Escherichia coli* is an integral constituent of the mammalian microflora, with several pathotypes of *E. coli* implicated in the development of enteric and extraintestinal diseases such as diarrhea, sepsis, meningitis, and urinary tract infections (224). Among the enteric *E. coli*-associated diseases, there are at least six different categories: enterotoxigenic *E.coli*, enteropathogenic *E. coli*, enterohemorrhagic *E. coli*, enteroaggregative *E. coli*, enteroinvasive *E. coli*, and diffusely adherent *E. coli* (225). Due to widespread antibiotic resistance of *E.coli* (226), IgY may serve as an alternative method to neutralize virulent *E. coli* in food, animals, and humans.

The major factor in the pathogenicity of *E. coli* is production of Shiga-like toxin. Numerous assays using IgY have been developed to detect the presence of Shiga-toxin-producing enterohemorrhagic *E.coli*. For instance, an ELISA assay involving IgY against *E.coli* O157:H7 was able to detect as little as 40 CFU/ml of E. coli O157:H7, suggesting that such assays can be used for detecting foodborne pathogens (227). Furthermore, because Shiga-toxin is uniformly expressed by all enterohemorrhagic *E. coli*, IgY can be used to detect different serotypes and variants of Shiga-toxin-producing *E. coli* (228). Similarly, toxin-specific IgY has also been used to detect and neutralize heat-labile toxin produced by enterotoxigenic *E. coli* (229).

Chicken or ostrich IgY against *E. coli* O157:H7 and O78:K80 inhibited bacterial growth in liquid medium (227, 230–232). The benefit of IgY given by intramuscular injection as prophylaxis against *E. coli*-associated respiratory, enteric, and septicemic diseases has been demonstrated in young broiler chicks (233). In piglets infected with enterotoxigenic *E. coli*, specific IgY decreased the severity of diarrhea (234) when encapsulated in chitosan-alginate microcapsules or hydrogel-carbon nanotube composites (235, 236), suggesting that this approach may be especially useful to neutralize *E. coli* IgY in food and veterinary settings.

A randomized, double-blind, placebo-controlled trial evaluated 301 Guatemalan children (154 intervention and 147 placebo) with acute non-bloody diarrhea who received PTM202 (combined IgY specifically targets rotavirus, enterotoxigenic *E. coli*, Shiga toxin-positive *E. coli*, and salmonella) or placebo for 3 days (237). PTM202 led to a reduction in duration of diarrhea among children whose diarrheal stool at enrollment contained one or more PTM202-targeted organisms. No adverse events were reported.

#### Detection and Treatment of *Staphylococcus aureus*

*Staphylococcus aureus* is a pathogenic bacterial strain that causes food poisoning, toxic shock syndrome, endocarditis, sepsis, soft tissue infections, and in-hospital infections (238, 239). Although *S. aureus* is normally found in mammals and 30-50% of humans, it remains a dangerous pathogen due to its endotoxicity, virulence, invasiveness, and antibiotic resistance (240). *S. aureus* has increasingly displayed resistance to common antibiotic treatments such as methicillin and vancomycin (241, 242) and antimicrobial-resistant strains are now detected in the community, not just in healthcare settings (243, 244). Stronger measures to contain *S. aureus* infections, as well as alternative and combination treatments, are now promoted to combat increasing antibiotic resistance.

*S. aureus* is commonly found in food samples. IgY has been employed as a method to detect the bacterium, as well as its associated proteins and toxins to prevent *S. aureus*-induced food poisoning. IgY against *S. aureus* or its endotoxins has been used in sandwich ELISAs, immuncapture polymerase chain reaction ELISAs, lateral flow devices, immunopillar chips, immunochromatography, and fluorescence resonance energy transfer assays (245–252). The success of these approaches is considered due to IgY’s lack of reactivity with protein A of *S.aureus*, which often results in false positives results in other assays (253). In vitro, specific IgY had high binding specificity and inhibited bacterial growth in culture, possibly by interrupting interactions with surface antigens (254). IgY generated against *S.aureus* also caused agglutination of the bacterium, and did not show cross-reactivity with other bacterial strains such as *Streptococcus epidermidis*, *Escherichia coli*, and *Pseudomonas aeruginosa* that are commonly found in milk (255).

*S. aureus* is also implicated in ruminant mastitis, which affects the quality and quantity of milk produced, as well as the health of infected animals (256). Although there are antibiotic treatments and vaccines available, they are not fully effective and *S. aureus*-associated infections often recur (257). IgY produced against *S. aureus* was highly specific to mastitis-causing strains, enhanced the phagocytic activity of milk macrophages (258), and reduced bacterial growth in culture (259). In addition, specific IgY blocked the internalization and infection of bovine mammary epithelial cells by *S.aureus in vitro* (260). At a concentration of 20 mg/ml, IgY anti-*S.aureus* infused by insertion into the teat canal decreased somatic cell and bacterial counts, while curing most experimentally challenged lactating cows (258). Similar cure rates were also observed in challenged buffaloes with mastitis, and specific IgY administered through intramammary infusions at a dose rate of 20 mg/ml improved milk yield (261).

The anterior nare of the nose is the most frequent carriage site for *S. aureus* (262). When the nares are treated topically to eliminate nasal carriage, in most cases the organism also disappears from other body areas (262). Anti-*S. aureus* IgY given intranasally may be of special interest in the treatment of this pathogen, including for the growing threat of methicillin-resistant *S. aureus* (263).

#### Detection and Treatment of *Aeromonas*

*Aeromonas* is commonly found in aquatic environments and the microflora of animals and humans. However, certain strains of *Aeromonas* have been implicated in the development of sepsis and gastroenteritis in humans (264), as well as fish, other animals, and environmental reservoirs (265). Notably, almost all subspecies of *Aeromonas* express strong resistance to beta-lactam antibiotics such as penicillin, ampicillin, and carbenicillin, which has led to the pursuit of alternative and combination antimicrobial therapies (266).

IgY-specific antibodies have been investigated as an alternative method to diagnose *Aeromonas*-diseased aquatic animals. IgY produced against *Aeromonas hydrophila* detected *A. hydrophila* in tissues and phagocytes in infected Nile tilapia (267), neutralized bacterial adhesins and toxins released by the bacteria, promoted agglutination, inhibited bacterial growth, and enhanced the phagocytic activity of infected Nile tilapia, blunt snout bream (268, 269), and polyploid gibel carp (269, 270). When added to rearing water, specific IgY eliminated the development of skin ulcers, as well as transmission of the infection between different fish (271).

### Skin-Related Infections

#### Treatment of Propionibacterium

Acne vulgaris is a skin condition that affects most humans at some time and is thought to be caused by multiple factors, including increased sebaceous gland sebum production, hormones, cytokines, nutrition, and bacteria such as *Propionibacterium acnes* (272). Because of rising antibiotic resistance, IgY has also been proposed as a cost-effective alternative to antimicrobial treatments of acne. IgY anti-*P. acnes* inhibited growth of *P. acnes* colonies as well as biofilm development by preventing bacterial adhesion (273).

## Diagnosis and Treatment of Fungal Infections

### Treatment of Candida albicans

Typically, *Candida albicans* is a normal, commensal member of the human microbiome (274). However, *C. albicans* can also express virulence factors that can cause candidiasis infections of the mucosal membranes of the oral cavity, esophagus, gastrointestinal system, vagina, vascular system, and skin, as well as serious and life-threatening pneumonia and systemic infections (275). Because of limited antifungal therapeutics, virulence, and the development of resistance to existing drugs (276), novel approaches to treatment, such as the use of IgY antibodies, have been investigated. IgY generated against *C. albican*s inhibited biofilm formation and growth in liquid media (277), as well as adhesion of *C. albicans* to denture-based material (272), human oral epithelial cells (277, 278), and human pharyngeal carcinoma cells (274). In addition, cross-reactivity to other *Candida* subspecies such as *C. glabrata* was observed, suggesting that anti-*C. albicans* IgY may also be used to prevent the dissemination of different *Candida* strains (277, 279). In mouse models, orally administered treatments of a gel containing 0.5 g of IgY against *C. albicans* (280) or a 20 mg specific IgY/ml phosphate-buffered saline solution (276) protected challenged animals and prevented *C. albicans* colonization and the subsequent development of kidney and tongue lesions. Because there was no correlation observed between drug-resistant strains of *C. albicans* and the growth-inhibition ability of anti-*C. albicans* IgY (278), specific IgY may warrant evaluation as a novel therapeutic for fungal infections in humans.

## Diagnosis and Treatment of Parasitic Diseases

### Treatment of *Trypanosoma*

Infection by the protozoan parasite *Trypanosoma cruzi* results in Chagas disease, which is transmitted to animals and humans by insect vectors that are found only in the Americas. Chronic infection can lead to heart and gastrointestinal disease that can be life-threatening (281). Vaccines developed against *T. cruzi* are only partially protective since defined antigens must be used to prevent the occurrence of cryptic infections (282). Furthermore, drugs approved for the treatment of Chagas disease can have toxic, mutagenic, and other adverse side effects (283). Since no cytotoxic or proliferative effects were observed on mononuclear and VERO cells *in vitro* when treated with IgY against *T. cruzi*, specific IgY has been considered as a possible therapeutic for Chagas disease (284). In a mouse model, anti-*T. cruzi* IgY administered prophylactically at 50 mg/kg reduced parasitemia post-challenge and prevented the development of cardiac lesions by amastigotes. These same effects were also observed with the therapeutic administration of 50 mg/kg of IgY, which also improved the immune response by preventing an increase in activity of E-NTPDase and E- ADA activities in the splenic lymphocytes of the animals (285).

Another member of the *Trypanosoma* family, *T. evansi*, infects a wide range of domesticated livestock worldwide (286), causing anemia (287). One case of *T. evansi* infection in humans has also been documented, possibly by blood transmission from an infected animal (288). Current strategies for controlling the dissemination of *T. evansi* include herd culling (289) and chemical therapy of infected animals, although this latter approach has limited use due to high toxicity and the development of drug-resistant strains (290). In contrast, no significant cytotoxic or genotypic toxicity was observed when IgY anti-*T. evansi* was used to treat peripheral blood samples, although an increase in cell viability and lymphocyte proliferation was observed when a concentration of 10 mg/ml of specific IgY was used (291). In vivo, specific IgY against *T. evansi* administered intraperitoneally at a dose of 10 mg/kg increased the longevity and survival of infected animals (292).

### Detection and Treatment of *Cryptosporidiosis*

Several members of the *Cryptosporidium* family are implicated in the development of cryptosporidiosis, an intestinal infection that causes diarrhea and, less commonly, pneumonia in humans (293). There are no therapies to fully treat cryptosporidiosis or prevent the infection in humans and animals (294) although hydration and passive immunization through the administration of monoclonal antibodies (295), nitazoxanide, or hyperimmune bovine colostrum have limited efficacy (296, 297). IgY has been explored as a treatment against *Cryptosporidium* infection. In vitro, IgY antibodies generated against *C. parvum* oocyst antigens were highly specific (298), decreased binding of the parasite to Caco-2 cells, and blocked the vitality of *C. parvum* (299). However, in a severe combined immunodeficiency mouse model, treatment using feed containing 25% specific IgY powder and a 20% specific IgY solution was only capable of partially reducing oocyst shedding in challenged animals, and was unable to eliminate infection (299). Similarly, IgY against the P23 protein in *C. parvum* also has high specificity for the parasite (300, 301). Using a mouse model, the anti-P23 IgY reduced oocyst shedding by 70% (300). Specific IgY against the GP60 glycoprotein in *Cryptosporidium hominis*, another strain implicated in cryptosporidiosis, was also found to specifically bind to the antigen, as well as the parasite (302). The high specificity and protective effectiveness of these antibodies suggest that IgY warrants evaluation as a novel diagnostic test for cryptosporidiosis and a passive immunization treatment in immunocompromised individuals.

### Treatment of *Eimeria*

The *Eimeria* family of parasites is responsible for the development of avian coccidiosis, a severe intestinal disease with varying rates of morbidity and mortality (303). Because of the significant economic effect that coccidiosis has on the poultry industry, coccidiostat drugs have been undertaken. Unfortunately, numerous *Eimeria* strains bear multiple resistance to these anticoccidials (304). Thus, other measures such as live attenuated vaccines (303) and IgY antibodies against *Eimeria* have been explored (305).

Using maternal immunization against the three major species of *Eimeria* that cause coccidiosis (*E. tenella*, *E. acervulina*, and *E. maxima*), Wallach et al. demonstrated that IgY protected offspring chicks up to 3 weeks of age (306). By strongly blocking the exponential rise in oocyst numbers, this was expected to also protect against the pathogenic effects of the disease for their entire 5-7-week growth period. Studies have occurred on a very large commercial scale where millions of hens and their offspring chicks were vaccinated on 4 continents (307). Maternal immunity can also lead to results similar or even better than that achieved using the best anticoccidial drugs (307).

In young broiler chicks challenged with *E. acervulina* oocysts, specific IgY against multiple strains of *Eimeria* decreased the number of oocysts in fecal matter and increased the body weight of treated animals (308). Similar improvements, as well as decreased intestinal lesions, were also observed in chicks challenged with *E. tenella* or *E. maxima* that were treated with Supracox, a commercially available egg yolk powder containing specific IgY against oocysts of three different *Eimeria* strains (309). With an IgY treatment generated against five different species of *Eimeria*, chicks challenged with *E. tenella* had reduced mortality, increased body weight gain, reduced oocyst shedding, reduced caecal lesion score, and increased anticoccidial index (310). IgY generated against a recombinant protein (3-1E) from *E. acervulina* merozoites also improved body weight gain and decreased oocyst production in chickens infected with *E. acervulina* or *E. tenella* (311). The Fc fragments of IgY have also been coupled to stable transgenic *Eimeria mitis*, which decreased oocyst output in challenged animals (312) and suggests that the method of expressing Fc-fused exogenous antigens on transgenic *Eimeria* might be explored as a more efficient vaccine.

## Principles Regarding the Clinical Use of IgY

### Physicochemical Properties of Chicken IgY Contribute to Safety

IgY consumed orally is considered to be GRAS (“Generally Recognized as Safe”) by the U.S. Food and Drug Administration. At a biochemical level, this may be because IgY, unlike IgG, does not have a hinge region but contains a short region between the Fa and Fc segments of the antibody’s stock (313). This short region is rich in proline and glycine residues, allowing only limited flexibility of its Fab. Due to this difference, IgY is unable to bind to mammalian Fc receptors and therefore does not readily cause T cell activation (314). In addition, because of its unique Fc characteristics, IgY does not cause the false-positive results or aggregation in immunoassays often seen with use of monoclonal and polyclonal mammalian antibodies (5). When used as a therapeutic, IgY - unlike mammalian antibodies - also cannot activate the mammalian complement cascade that may cause cell lysis (314), even after repetitive subcutaneous injections *in vivo*. However, IgY used as a repetitive parenteral product is unlikely due to risk of an adverse immune response.

In humans, controlled clinical trials have shown that IgY has a favorable safety profile with its known nonparenteral administration. Continuous prophylactic treatment by a daily mouth rinse with specific IgY against *P. aeruginosa* in 17 patients with cystic fibrosis for up to 12 years (114 patient-years) significantly reduced pulmonary *P. aeruginosa* infections compared with 23 cystic fibrosis control patients, with no adverse events (119, 315). Use of oral anti-*P. aeruginosa IgY* gargling solution by 83 patients with cystic fibrosis for a median of 26.3 months resulted in a similar number and incidence of adverse events compared with 81 patients receiving placebo (120).

Additional clinical studies showing favorable safety of IgY given orally include treatment with a gel containing anti-Candida IgY (316), treatment with lozenges containing anti-*P. gingivalis* IgY (317), ingestion of anti-rotavirus IgY in pediatric patients with rotavirus diarrhea (63, 67), and ingesting yogurt containing anti-*H. pylori* IgY (167). Parenteral administration of IgY has not been evaluated but may be of value as a single injection in situations such as the need for antivenom treatment. IgY-based antivenom given parenterally resulted in complete protection in animal models of lethal venomous bites and stings (318–321).

### Stability

Due to its physical and biochemical properties, IgY is protected from inactivation down to pH 3 (8), heat up to 60°C (156), and proteolytic enzymes (313). At a pH lower than 3, such as in the gastrointestinal tract, inactivation of IgY is similar to other immunoglobulins but can be minimized by encapsulation (322). IgY can also be degraded by stomach protease and pepsin, thereby reducing IgY’s efficacy in the treatment of gastrointestinal diseases (4). However, a variety of formulation ingredients can increase IgY stability. For example, a 50% solution of sorbitol is a polyol that is commonly used to improve protein stability. Sorbitol at this concentration is sufficient to prevent the pH-dependent inactivation of IgY by strengthening the hydrophobic interactions and by surrounding and preventing the exposure of its aromatic or carboxylic amino acids, the sites of pepsin proteolysis (323).

Coupling of polyethylene-glycol improves the heat stability of IgG, and therefore may also increase the heat resistance of IgY (324). High concentrations of sucrose also had a stabilizing effect on IgY: in a 50% sucrose solution, the activity of IgY was completely retained even when heated to 80°C (325). Furthermore, the encapsulation of IgY in liposomes renders IgY resistant to acidic conditions and pepsin (326, 327). Orally administered chitosan-alginate microcapsules also protected premature degradation and improved bioactivity and targeting of IgY to microorganisms in the lower intestinal tract of neonatal and early-weaned piglets (235).

### Production Process

IgY may provide a low-cost and fast method for the development of prophylactic immunization strategies. The phylogenetic relationship between birds and mammals not only permits the adaptation of IgY as an alternative to mammalian antibodies for clinical use, but also increases sensitivity of IgY to human antigens for diagnostics. Thus, immunization of laying hens twice with the antigen of interest is sufficient to produce a humoral response that results in the production of large amounts of specific IgY molecules in their eggs for several months. Typically, older hens produce a higher titer compared with younger chickens (328). As mentioned previously, IgY collection does not require bleeding of the immunized laying hens or their progeny, thus reducing animal distress and other ethical considerations.

A typical egg of an immunized hen contains about 100 mg of IgY and a single immunized hen lays approximately 325 eggs per year, resulting in a total production yield of up to 40 g of IgY per hen per year (329). We estimate that each egg can produce approximately 10 doses of treatment/person, varying with the indication. Two to 10% of the IgY found in eggs of immunized commercial hens is specific to the antigen of interest (330). A much higher amount, 500 mg of IgY per egg, is produced in specific pathogen-free hens, hence the percentage of specific antibodies is likely higher; specific IgY levels can be maintained without further inoculation of the laying hen for up to half a year (230). IgY can be purified *via* a variety of nontoxic methods, including water dilution and low pH-induced precipitation, as well as polyethylene glycol-, dextran sulfate- and xanthan gum-induced precipitations (331) or NaCl extraction (332). Therefore, in terms of environmental impact, IgY can be efficiently collected and processed on an industrial scale without the usage of harsh chemicals and the production of toxic byproducts (8). Very large quantities of IgY antibodies can be harvested from the eggs laid by a single immunized hen, and the overall yield is comparable to the amount that can be collected from large mammals such as goats and cows, and 18-times higher than what is collected from rabbits (9).

## Concluding Remarks and Future Perspectives

Use of avian IgY antibodies has important implications for a wide range of human and veterinary clinical applications. As a diagnostic tool, the inflexibility of IgY’s structure prevents binding to rheumatoid factor, which is found in mammalian samples. Because of this advantage conferred by IgY’s structure, the use of IgY antibodies in immunoassays may result in less background noise, fewer false positives, and decreased aggregation of antibodies, which are common issues observed with both monoclonal and polyclonal mammalian antibodies. Furthermore, IgY antibodies have also shown high binding specificity and low cross-reactivity with other antigens comparable to current industry standards and may have value in a variety of applications to detect pathogens.

IgY has been explored as a prophylactic agent with the potential to neutralize pathogens *in vivo*. IgY antibodies can be developed into highly stable and concentrated products that can be evaluated under controlled settings in human and veterinary subjects, with the route depending on the intended target of prophylaxis. Nonparenterally administered IgY products, such as oral ingestibles, nasal sprays, and nasal drops, may provide widespread protection against pathogens that can colonize, infect, or damage the gastrointestinal and respiratory tracts. Neutralizing IgY antibodies may also warrant evaluation to target specific pathogens circulating in the bloodstream or localized in a specific area. Because IgY administration before infection has been demonstrated to have significant protective effects *in vivo* in animal models, evaluation of its use as prophylactic therapy in humans may be of special interest, including for SARS-CoV-2 in context of the current COVID-19 pandemic. Besides use in passive immunotherapy, IgY antibodies have also shown promise as a potential therapeutic agent for a wide spectrum of clinical applications. Specific IgY has detected and neutralized both surface and internal pathogenic antigens when administered after infection or consumption in multiple preclinical models. In most of these applications, IgY is not curative and has a greater therapeutic benefit with greater protection when used as prophylactic treatment or in conjunction to supplement existing standard treatments.

Limitations of IgY include possible host anti-IgY antibody responses. Parenteral administration has potential for serum sickness, an immune-complex-mediated hypersensitivity reaction. IgY immunogenicity has been tested in a pig model, which revealed that an anti-IgY antibody response was induced upon both local and systemic routes of administration of IgY (333, 334). Further safety studies are necessary to explore the potential of an antigenic or allergic response in both human and veterinary applications of IgY. Because of the phylogenetic distance between IgY and mammalian immunoglobulins, IgY is unable to bind to and activate Fc receptors, rheumatoid factors, and the mammalian complement system. Acute responses through these mechanisms would be unexpected.

Lack of intellectual property for IgY has hampered the commercial development of therapeutics by industry. Despite this, the potential of IgY to address a wide range of common and fatal diseases in both farm animals and humans is gaining interest (335–338) and warrants further exploration against infectious agents, including those associated with pandemics. For example, the distribution of COVID-19 vaccines with outstanding safety and efficacy profiles remains limited, with an estimated 5.5% of the world population fully vaccinated as of June 1, 2121 (339). A key bottleneck in mRNA (COVID-19) vaccine manufacturing is a global shortage of essential components (such as nucleotides, enzymes, and lipids) (340), which has lessened production of the estimated 11 billion doses needed to fully vaccinate 70% of the world’s population – the figure assumed needed to reach herd immunity. This shortage especially impacts low- and lower-middle-income countries (340). In contrast, within 6 weeks of identifying an epidemic or pandemic-causing virus, millions of egg-laying hens immunized throughout the world can provide billions of doses of drug substance, as each egg can produce many doses.

## Author Contributions

Wrote or contributed to the writing of the manuscript: LL, KS, MW, LF, and DM-R. All authors contributed to the article and approved the submitted version.

## Funding

The authors would like to thank SPARK at Stanford and the Stanford ChEM-H and the Innovative Medicines Accelerator Award, May 2020 COVID-19 Response: Drug and Vaccine Prototyping Seed Grant Program for support of the project.

## Conflict of Interest

The authors declare that the research was conducted in the absence of any commercial or financial relationships that could be construed as a potential conflict of interest.

## Abbreviations

AE, adverse event; BLV, bovine leukemia virus; CD, Clostridium difficile; COVID-19, coronavirus disease 2019; ELISA, enzyme-linked immunosorbent assay; IgG, immunoglobulin G; IgY, immunoglobulin Y; HBV, hepatitis B virus; HPS, hantavirus pulmonary syndrome; HPU, *H. pylori* urease; IBDV, infectious bursal disease virus; MERS, Middle East Respiratory Syndrome; MERS-CoV, Middle East respiratory syndrome coronavirus; MBTC, Mycobacterium tuberculosis; SARS, Severe Acute Respiratory Syndrome; SARS-CoV, SARS-associated coronavirus; SARS-CoV-2, severe acute respiratory syndrome coronavirus 2.
